# Supplementary material for: N-cadherin facilitates trigeminal sensory neuron outgrowth and target tissue innervation
Source: Development. 2025 May 1;152(9):dev204369. doi: 10.1242/dev.204369 (PMC12070061; doi:10.1242/dev.204369)
Supplement: Supplementary information [file develop-152-204369-s1.pdf]

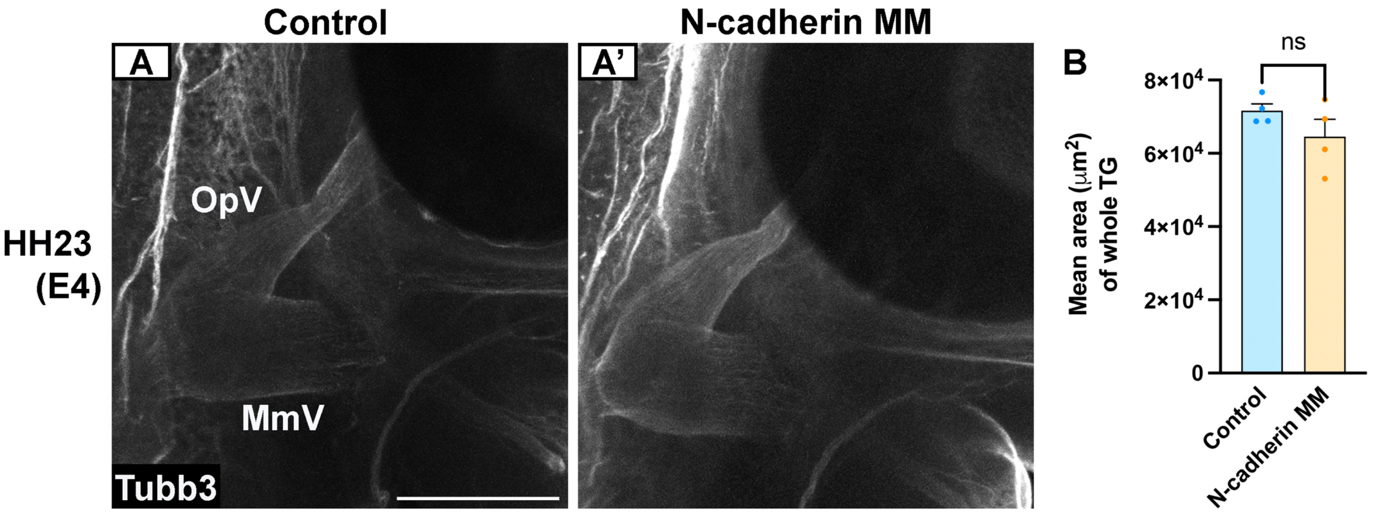

Fig. S1. N-cadherin mismatch control MO (MM)-treated embryos show normal trigeminal ganglion morphology. (A-A') Representative images of the trigeminal ganglion after Tubb3 immunostaining (E4.5/HH23-25, (n=4)). Scale bar in (A) is 250 µm and applies to (A'). (B) Mean whole trigeminal ganglia area ± SEM for contralateral control and N-cadherin MM sides of embryos. Statistical significance was determined via an unpaired t-test. Abbreviations: TG: trigeminal ganglion; OpV: ophthalmic; MmV: maxillomandibular; MM: mismatch control; MO: morpholino; ns: not significant.

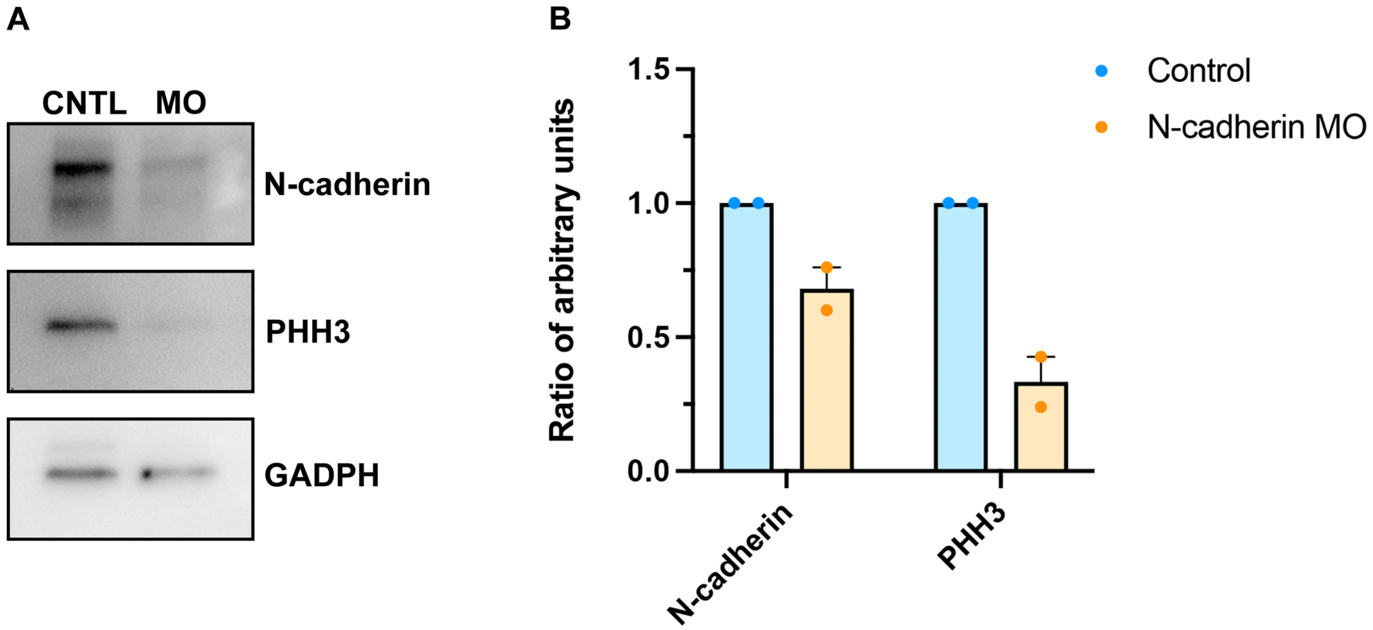

Fig. S2. N-cadherin knockdown in placode cells does not increase cell proliferation at later developmental stages. (A) Immunoblots of E6.5/HH28-30 electroporated (MO) and contralateral (CNTL) trigeminal OpV lobes for N-cadherin, phospho-histone H3 (PHH3), and GAPDH (n=2). (B) Protein levels of N-cadherin and phospho-histone H3 normalized against GAPDH loading control, presented as a fraction of total signal from contralateral control OpV lobes.

Development • Supplementary information

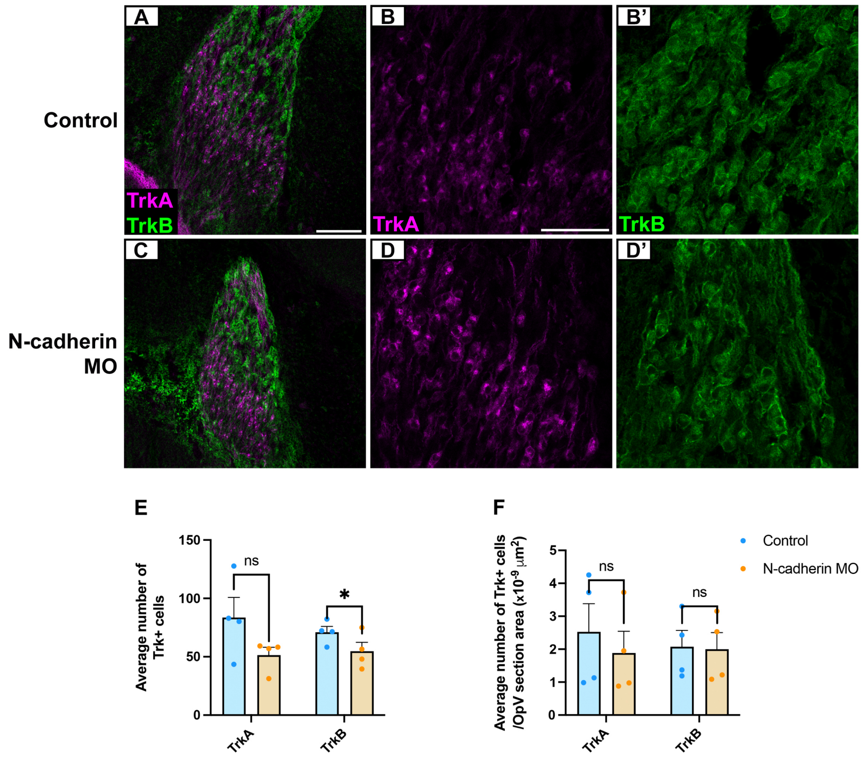

Fig. S3. N-cadherin depletion from placode cells does not alter subpopulations of sensory neurons within the trigeminal ganglion. (A-D') Sagittal serial sections through the OpV lobe of the trigeminal ganglion (contralateral control (A-B') and N- cadherin MO-electroporated side (C-D')) from representative E5.5/HH26-27 (n=4) embryos with TrkA and TrkB immunostaining. Images of TrkA and TrkB serial sections were merged to generate representative images. Scale bar in (A) is 100 μm and applies to (C); scale bar in (B) is 50 μm and applies to (B, D-D'). (E) Average number of TrkA- or TrkB-positive OpV neurons ± SEM after N- cadherin MO electroporation. (F) Average number of TrkA- or TrkB-positive OpV neurons normalized against OpV lobe area ± SEM. Statistical significance was determined via paired t-tests (E, F). (*) $p<0.05$.

Development • Supplementary information

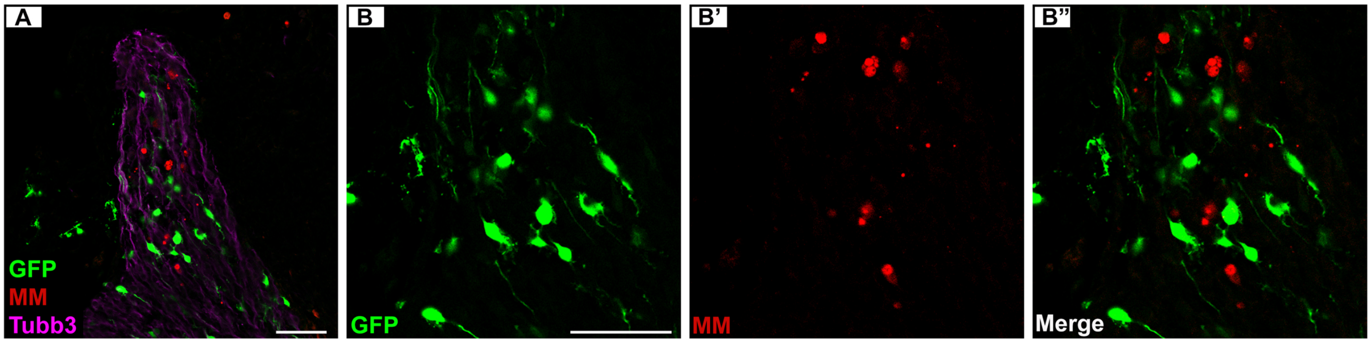

Fig. S4. Sequential electroporation of neural crest cells and placode cells exclusively labels distinct cell populations. Representative images of an HH25/E4.5 embryo sequentially electroporated with PiggyBacGFP plus PBase (neural crest cells) and N-cadherin MM (placode cells), followed by Tubb3 and GFP immunostaining. Scale bar in (A) is 100 μm and scale bar in (B) is 50 μm and applies to (B'-B'').

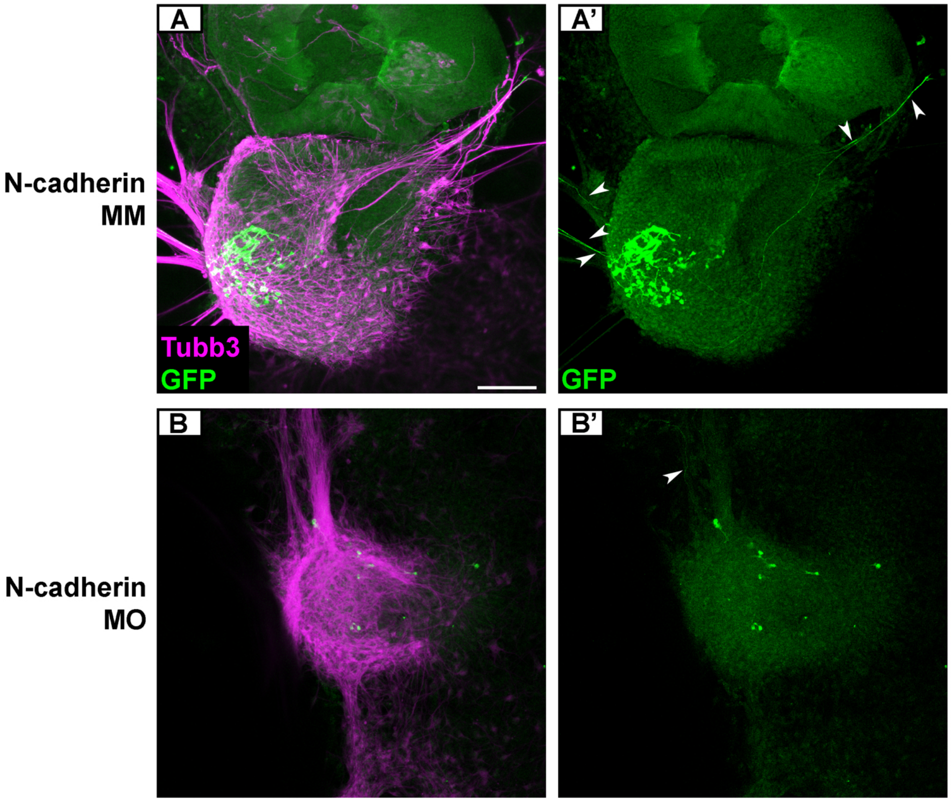

Fig. S5. N-cadherin MO-treated explants qualitatively exhibit less neural crest-derived axon outgrowth. (A-B') Representative images of sequentially-electroporated trigeminal explants with neural crest cells co-electroporated with PiggyBacGFP plus PBase, and placode cells electroporated with N-cadherin MM (A-A') or N-cadherin MO (B-B'), followed by GFP and Tubb3 immunostaining. Arrowheads in (A') and (B') point to neural crest-derived axons. Scale bar in (A) is 100 μm and applies to all images.
