# Supplementary material for: N-cadherin facilitates trigeminal sensory neuron outgrowth and target tissue innervation
Source: Development. 2025 May 1;152(9):dev204369. doi: 10.1242/dev.204369 (PMC12070061; doi:10.1242/dev.204369)
Supplement: Supplementary file 1 [file develop-152-204369_review_history.pdf]

N-cadherin facilitates trigeminal sensory neuron outgrowth and target tissue innervation

Caroline A. Halmi, Carrie E. Leonard, Alec T. McIntosh and Lisa A. Taneyhill
DOI: 10.1242/dev.204369

Editor: Debra L. Silver

Review timeline

Original submission:	2 September 2024
Editorial decision:	24 October 2024
First revision received:	3 March 2025
Editorial decision:	21 March 2025
Second revision received:	25 March 2025
Accepted:	1 April 2025

Original submission

First decision letter

MS ID#: dev.204369

MS TITLE: N-cadherin facilitates trigeminal sensory neuron outgrowth and target tissue innervation

AUTHORS: Caroline A. Halmi; Carrie E. Leonard; Alec T. McIntosh; Lisa A. Taneyhill

Thank you for your patience as we only recently received the third reviews. I have now received all the referees' reports on the above manuscript, and have reached a decision. The referees' comments are appended below, or you can access them online: please go to:

As you will see, the referees express considerable interest in your work, but have some significant criticisms and recommend a substantial revision of your manuscript before we can consider publication. In particular, you will note that Reviewer 1 suggests several new experiments to clarify mechanisms, address possible confusion in the data, and to address outstanding concerns raised by the study . While it may not be necessary to address all of reviewer 1's concerns, this reviewer does feel completion of some of these is needed in order for your study to make a sufficient conceptual advance. Thus, please complete those experiments which will further explain the mechanism of action of N Cadherin and support conclusions of your study. You will also see concerns raised by other 2 reviewers, mostly minor.

If you are able to revise the manuscript along the lines suggested, which may involve further experiments, I will be happy receive a revised version of the manuscript. Your revised paper will be re-reviewed by one or more of the original referees, and acceptance of your manuscript will depend on your addressing satisfactorily the reviewers' major concerns. Please also note that Development will normally permit only one round of major revision. If it would be helpful, you are welcome to contact us to discuss your revision in greater detail. Please send us a point-by-point response indicating your plans for addressing the referees' comments, and we will look over this and provide further guidance.

Please attend to all of the reviewers' comments and ensure that you clearly highlight all changes made in the revised manuscript. Please avoid using 'Tracked changes' in Word files as these are lost in PDF conversion. I should be grateful if you would also provide a point-by-point response detailing

© 2025. Published by The Company of Biologists under the terms of the Creative Commons Attribution License (https://creativecommons.org/licenses/by/4.0/).

how you have dealt with the points raised by the reviewers in the 'Response to Reviewers' box. If you do not agree with any of their criticisms or suggestions please explain clearly why this is so.

Reviewer 1

Advance summary and potential significance to field

Halmi et al uses the chick model to determine the function of N-cadherin in trigeminal nerve outgrowth. Specifically, the authors tested the role of N-cadherin in placodal neurons axon outgrowth and target innervation. They then utilize knockdown followed by immunofluorescence and axon guidance assays to show that N-cadherin knockdown in placodal cells affects neural crest derived neurons, neurite number and branching. Placodal neurons have reduced axon outgrowth as well. The manuscript advances our knowledge in the field by adding outgrowth as a role for N-cadherin. It is well written and illustrated. However, more can be done to determine the mechanism of action of N-cadherin. I have added some suggestions that would strengthen the manuscript.

Comments for the author

1. It is interesting that the recovery occurs at the later time points. The authors suggest two interesting possibilities, including that the MO will not stay active. Is there a way to inject at later timepoints to test this idea? Another possibility is that after the knockdown there is an increase in cell proliferation. It is worth testing to determine the effect on proliferation in these embryos.
2. Further, in looking at the cell death images, two questions here. The ectoderm is highly labeled with the MO injection suggesting that when these experiments are done that there is more than the trigeminal placode being knockdown with N-cadherin. Could the knockdown in other cranial ganglia affect the innervation pattern? In addition, most if not all of the cell death is in non-MO labeled cells. How do the authors explain this result as one might expect some death in the labeled cells that then results in a non-autonomous defect.
3. From the images of the nasal branches in Figure 2 it also looks like there is a defect in fasciculation especially in the lateral branches because the look thickened. Which seems a bit counterintuitive since if there is less adhesion, it would suggest less fasciculation. Is there a way to quantify this phenotype further? What is the interpretation of this?
4. The experiment with the function blocking N-cadherin antibody is a very interesting approach. However I feel like there is a missed opportunity to label and knockdown from both the neural crest and the placode is the same culture set up. This would test the role of N-cadherin but also allow for interactions of placode with neural crest. It also seems to me that the N-cadherin knockdown has more Tubb3 expression (Figure 4D, Supp Figure 2) which is inconsistent with the previous results presented here.
5. It would be interesting to explore further which neuronal subtypes are affected by N-cadherin knockdown. Are specific classes of sensory neurons affected more than others? Antibody staining for specific receptors would be able to answer this question more directly.
6. More questions arise from the end of the manuscript. Is this phenotype from N-cadherin knockdown only based on adhesion between neurons or fate of the cells? Are their changes in the adhesion strength or tension levels in the ganglia? Or is B-catenin or p120 or cytoskeleton affected in a non-autonomous way? Gene expression changes? Or as suggested is knockdown affecting pioneer axons? How could this be tested? While I understand all of these experiments cannot be done, adding some additional information would help strengthen the manuscript.

Minor:
1. Measuring RFP signal intensity is not best way to quantify protein levels given the instability of fluorescent proteins, if no other measure accompanies it ie qRT-PCR or quantitative RNA in situ or protein measures.

Reviewer 2

Advance summary and potential significance to field

© 2025. Published by The Company of Biologists under the terms of the Creative Commons Attribution License (https://creativecommons.org/licenses/by/4.0/).

This well written paper uses elegant experimental approaches in the chick to explore the role of N-Cadherin in gangliogenesis and neurite outgrowth of the trigeminal sensory system. While a role for N-cad in initial assembly of the ganglion has been previously explored, this study delves into later axon outgrowth and target innervation, revealing an important later role for N-cadherin. The most important aspects of the work are the exploration of interactions between neural crest and placode derived neurons, and the elegant demonstration of a non-cell autonomous effect of N-Cad depletion on neurite outgrowth from neural crest derived neurons. There is general interest in placode/neural crest interactions, and this study adds another important facet to our understanding of that relationship.

Comments for the author

The experiment shown in Fig. 4 takes an elegant approach to allow the separation of analysis of neural crest-derived neurons from the manipulation of placode-derived neurons. This is the key experiment that shows non cell-autonomous action of NCad, so it is critical to confirm that the GFP+ve neural crest-derived neurons have not received any morpholino reagent (labeled in magenta). The authors should thus show a panel with the magenta channel alone so the reader can be confident the morpholino reagent did not spill into the neural crest area.

Fig. 6 shows that N-Cad blocking antibodies block axon outgrowth of placode-derived neurons in an explant system. The figure title accurately states that the majority of placodal neurons rely on N-cad for axon outgrowth. By contrast, the results section makes the claim that this is a cell autonomous effect. Given the technique used, these data (and others in the paper) support tissue level autonomy of function, but not necessarily cell level autonomy (i.e. at an individual cell by cell level). The text - including the abstract - should be re-written to reflect this.

The authors should briefly discuss the placode/crest interactions that occur in the Xenopus 'chase and run' study from Theveneau and Mayor as N-Cad is involved here too - albeit in a repulsive context.

Minor point
Fig. 3A is missing a scale bar

Reviewer 3

Advance summary and potential significance to field

This manuscript examines the role of N-cadherin during trigeminal ganglion development in the chick embryo. The neurons of this cranial ganglion are both placode- and neural crest-derived, with the placode-derived neurons being the first to send out axons. Taking advantage of the chick embryo, the authors demonstrate in vivo and in vitro that N-cadherin is required for axon outgrowth in both placode- and neural crest-derived neurons cell autonomously and non-cell autonomously. This work provides a significant conceptual advance to our understanding of gangliogenesis during early nervous system development because it establishes a sustained role for N-cadherin during multiple stages of trigeminal ganglion development, as well as provides evidence for the role of cell adhesion molecules in pioneer neurons.

Comments for the author

Major comments:

1. With regard to Figure 4, was there a nuclear label or some way for the authors to be certain they were only measuring from a single soma? Based on the size of the GFP cells in Fig. 4B', C versus Fig. 4D', E, if they are to the same scale (as suggested by the figure caption), it looks to this reviewer that the control might be two cells adhered to each other? The GFP in this panel also looks oversaturated, which is adding to this concern. Also, as the authors noted in Supplemental Fig. 2 that the control had many more GFP+ cells that also looked closer/adhered to each other, this is likely more of an issue for the control counts in Fig. 4. Can the authors either clarify how they ensured single cells were counted (if they did so) or re-analyze with the addition of a nuclear

© 2025. Published by The Company of Biologists under the terms of the Creative Commons Attribution License (https://creativecommons.org/licenses/by/4.0/).

label (or something to ensure single cells are being measured)? Given that the quantitative analyses performed are based on neurites from a single soma, it is critical to ensure the authors are accounting for single cell bodies, especially because this experiment supports the non-cell autonomous function the authors posit for N-cadherin.

2. With regard to Figure 5, the authors nicely demonstrate that the addition of a function-blocking N-cadherin antibody reduced axon density in explant culture, which they attribute to defects in axon outgrowth. However, there are no data showing what the 24h explants look like prior to addition of the antibodies. Without seeing analysis of axon density and/or length measurements prior to antibody addition, can the authors exclude the possibility that N-cadherin inhibition induces axon pruning, rather than just defects in axon outgrowth/branching? A representative image of the explant prior to antibody treatment and similar quantitative analyses added to the figure would clarify this point.

Minor comments:

1. Is Figure 3 missing the scale bar?
2. Figure 4B has black text on the image [”is this meant to be there? If so, it's difficult to read]
3. The asterisks on some of the graphs (Fig. 2C,H; Fig. 5E) are very small and difficult to see.

First revision

Author response to reviewers' comments

We wish to thank you and the reviewers for your consideration and comments regarding our submitted manuscript entitled “N-cadherin facilitates trigeminal sensory neuron outgrowth and target tissue innervation” (Manuscript ID#: dev.204369). We are pleased that the reviewers liked the manuscript, and as requested by them and yourself, we have revised the manuscript and provided additional clarification according to their comments. Changes made to the manuscript text are highlighted in yellow.

Reviewer 1

1. The Reviewer writes “it is interesting that the recovery occurs at the later time points. The authors suggest two interesting possibilities, including that the MO will not stay active. Is there a way to inject at later timepoints to test this idea? Another possibility is that after the knockdown there is an increase in cell proliferation. It is worth testing to determine the effect on proliferation in these embryos.”

We thank the Reviewer for suggesting this experiment of later MO injections, as it would allow us to test the idea of recovery due to loss of MO. Unfortunately, we are not able to target the MO via injection and electroporation at these later stages. Since placodal neurons are now aggregated and condensed with neural crest-derived neurons and glia to form the trigeminal ganglion and its nerves, we can no longer specifically target only placodal neurons.

To address the issue of cell proliferation, we have performed immunoblotting for phospho-histone H3 on lysate from ophthalmic (OpV) lobes dissected from N-cadherin knockdown and control trigeminal ganglia at a stage grouping where we see no change in trigeminal ganglion size. Through these experiments, we found reduced cell proliferation upon N-cadherin knockdown, indicating that the absence of phenotype is not due to an increase in cell division within this stage grouping. This result is consistent with what has been reported in the literature in that most OpV placode cells become post-mitotic upon their delamination from the surface ectoderm, such that little cell division is occurring (Begbie, 2002; Graham and Shimeld, 2013; McCabe et al., 2009). As only a neuronal marker (Tubb3) was used in these experiments, it is also possible that the balance of neural crest cell differentiation into neurons vs. glia was altered, leading to more of the former over the latter, and we cannot discern this in our experiments. Interestingly, we noted that N-cadherin levels were still reduced in the N-cadherin knockdown samples in this stage grouping, indicating that either the MO is still active

© 2025. Published by The Company of Biologists under the terms of the Creative Commons Attribution License (https://creativecommons.org/licenses/by/4.0/).

and/or that N-cadherin levels have not “recovered” and returned to “normal.” We thank the Reviewer for suggesting this experiment as it has strengthened the manuscript and provided additional insight into the trigeminal ganglion morphology phenotypes that we observe over developmental time.

2. The Reviewer writes “the ectoderm is highly labeled with the MO injection suggesting that when these experiments are done that there is more than the trigeminal placode being knockdown with N-cadherin. Could the knockdown in other cranial ganglia affect the innervation pattern? In addition, most if not all of the cell death is in non-MO labeled cells. How do the authors explain this result as one might expect some death in the labeled cells that then results in a non-autonomous defect.”

We appreciate this comment by the Reviewer, as it allows us to expand upon our electroporation method and our results for OpV. While we have targeted our electroporations to the surface ectoderm rostral to the otic vesicle, there is the possibility that we may have inadvertently electroporated the geniculate and/or petrosal placode, which give rise to sensory neurons of cranial nerves VII and IX, respectively, due to some MO diffusion. However, these placode-derived neurons are not present in the eye region but instead respectively innervate the anterior two-thirds of the tongue, and the posterior one-third of the tongue, middle ear, carotid body and sinus, and the Eustachian tube. Therefore, we do not expect any potential effects on these other populations of placode-derived neurons to impact trigeminal sensory neurons observed in OpV.

Regarding the apparent absence of cell death in labeled cells, it is important to note that the average number of apoptotic cells per section is quite low (see Figure 3). While some of the TUNEL-positive signal may be in MO-negative cells, it is also plausible that these TUNEL-positive cells once possessed MO or may just have lower levels of MO in them that are not visible with microscopy, since we have just acquired a “snapshot” of TUNEL staining through our static section images. To further address the issue of cell death, however, we have now examined earlier stages of development (HH13-15) when placodal neurons are delaminating and beginning to migrate to the trigeminal ganglionic anlage and intermix with undifferentiated neural crest cells. To this end, we performed section immunohistochemistry using cleaved caspase-3 to label apoptotic cells (results now included with Figure 3), together with Tubb3 (labels placodal neurons). Through these assays, we found no significant cell death occurring at these earlier stages, further supporting our assertion that cell death is not a main driver for the observed size differences across developmental stages.

3. The Reviewer indicates that “from the images of the nasal branches in Figure 2, it also looks like there is a defect in fasciculation, especially in the lateral branches because they look thickened. [This] seems a bit counterintuitive since if there is less adhesion, it would suggest less fasciculation. Is there a way to quantify this phenotype further? What is the interpretation of this?”

We thank the Reviewer for bringing this observation and subsequent interpretation to our attention. The images presented in our manuscript are Z-stacks, which could explain the apparent increased thickening in them. Unfortunately, we are not able to directly compare the bundle width of corresponding axons in N-cadherin MO and contralateral control trigeminal ganglia without tracing, which is not possible to do in our current images given the phenotypic changes that we observe (i.e., we cannot precisely pinpoint individual, respective axons in N-cadherin MO and contralateral control samples to make these comparisons). Therefore, we cannot determine whether fasciculation is increased in these images. However, based on the high statistically significant decrease observed in total bundle width in N-cadherin MO-treated trigeminal ganglia compared to control, and the fact that our images are Z-stacks, we do not believe there to be increased thickening/fasciculation upon N-cadherin knockdown.

4. The Reviewer writes that while “the experiment with the function blocking N-cadherin antibody is a very interesting approach, [I] feel like there is a missed opportunity to label and knockdown from both the neural crest and the placode is the same culture set up….[to] test the role of N-cadherin but also allow for interactions of placode with neural crest.”

We appreciate this suggested experiment by the Reviewer. We have now performed

© 2025. Published by The Company of Biologists under the terms of the Creative Commons Attribution License (https://creativecommons.org/licenses/by/4.0/).

sequential elecroporations, introducing PiggyBacGFP into neural crest cells followed by PiggyBacRFP into placode cells, reincubation of the embryo to HH13/14, dissection of the trigeminal ganglion, and culture *in vitro* with either the rat IgG control or the N-cadherin function-blocking antibody (anti-N-cadherin). Unfortunately, we were not able to observe RFP-labeled placode cells interacting with/adjacent to GFP- labeled neural crest cells in our explant cultures. This may be due to the absence of the *in vivo* environment in an *in vitro* culture system and/or the fact that we examined our cultures after two days vs. earlier. However, this new experiment allowed us to examine the impact of blocking N-cadherin on neural crest- derived neurons and, remarkably, we observed results similar to those obtained after knocking down N- cadherin in placode cells. These data indicate that N-cadherin-mediated adhesion is an important driver in neural crest-derived neurite formation and have enhanced the manuscript, so we thank the Reviewer for their encouragement to pursue this assay.

5. The Reviewer writes "it also seems to me that the N-cadherin knockdown has more Tubb3 expression (Figure 4D, Supp Figure 2) which is inconsistent with the previous results presented here."

In Figure 4, we performed trigeminal ganglion explants after sequential electroporation (neural crest cells with PiggyBacGFP, placode cells with N-cadherin MO or mismatch control (MM) MO), with Supplemental Figure 2 (now new Supplemental Figure 5) being the lower magnification version of this image. In these *in vitro* assays, we do not make any measurements of Tubb3 fluorescence. The "previous results" to which we think the Reviewer is referring are the *in vivo* measurements in Figures 1 and 2. In these experiments, we did not quantify Tubb3 fluorescence but rather measured the size of the whole trigeminal ganglion and OpV (Figure 1) and determined the bundle width, innervation field area, and number of axonal intersections (Figure 2). We apologize for any confusion in the text and hope the Reviewer is satisfied with this explanation.

6. The Reviewer writes "it would be interesting to explore further which neuronal subtypes are affected by N-cadherin knockdown."

We thank the Reviewer for this experimental suggestion. To address this, we have collected serial sections on slides through the forming OpV lobe at HH26-27 (when we see robust defects in trigeminal ganglion morphology) after N-cadherin knockdown and performed immunohistochemistry for TrkA and TrkB (since our Trk antibodies are made in rabbit, we cannot perform co-staining). These Trk receptors represent specific classes of sensory neurons (TrkA: nociceptors; TrkB: mechanoreceptors) in the trigeminal ganglion. We have also labeled these slides with antibodies to Tubb3 (pan-neuronal). By merging images from these serial sections, we found that N-cadherin knockdown in placode cells decreased the overall number of TrkB neurons, with no change in TrkA neuron number. However, when normalizing the mean cell number to the area of the OpV lobe (to account for the size difference of the OpV lobe after N-cadherin knockdown), we saw no difference in TrkA or TrkB neurons. This data is included as (new) Supplemental Figure 3. While it is possible these neurons would show differences later in development as they further specialize, we did not observe any changes at this timepoint.

7. The Reviewer asks about potential mechanisms by which N-cadherin knockdown in placode cells is affecting neural crest-derived neurons in a non-cell autonomous manner, such as effects on "β-catenin, p120-catenin or the cytoskeleton."

We appreciate the Reviewer pointing out potential ways by which N-cadherin depletion from placode cells could be affecting neural crest-derived neurons in a non-cell autonomous manner. To address this, we have performed sequential electroporations (neural crest cells: PiggyBacGFF; placode cells: N- cadherin MO or mismatch control (MM) MO) followed by trigeminal ganglion explant cultures and immunostaining for β-catenin. Our results reveal that neural crest-derived neurons exhibit decreased β-catenin levels, including at the membrane, in N-cadherin MO-treated explants (new Figure 4H-J). We thank the Reviewer for suggesting this experiment, as it has now provided a mechanism for how loss of N-cadherin in once cell type (placode cells) negatively impacts another cell type (neural crest-derived

© 2025. Published by The Company of Biologists under the terms of the Creative Commons Attribution License (https://creativecommons.org/licenses/by/4.0/).

neurons), strengthening the impact of the manuscript.

Minor comment

The Reviewer writes that "measuring RFP signal intensity is not the best way to quantify protein levels given the instability of fluorescent proteins [in the absence of another] measure [like] qRT-PCR."

We agree that examination of *RFP* transcript levels are needed to bolster our RFP signal intensity measurements. Therefore, we have conducted quantitative PCR for *RFP* on explants treated with either rat IgG control or anti-N-cadherin. Our data indicate that *RFP* transcript levels are not statistically significantly different between these treatments (new Figure 6F) and thus correlate with our RFP protein results. We thank for Reviewer for proposing this experiment to corroborate the RFP intensity measurements.

Reviewer 2

1. The Reviewer states that in the experiment shown in Figure 4, "it is critical to confirm that the GFP+ve neural crest-derived neurons have not received any morpholino [MO] reagent (labeled in magenta). The authors should thus show a panel with the magenta channel alone so the reader can be confident the [MO] reagent did not spill into the neural crest cell area."

We thank the Reviewer for bringing this to our attention. First, we apologize for the lack of clarity in the presentation of our results. The magenta signal represents Tubb3 immunoreactivity, not MO fluorescence, which we have now clarified in the figure legend and text. However, the Reviewer brings up a valid point to ensure the absence of MO in neural crest cells. To address this, we now include a section image from an embryo sequentially electroporated with PiggyBacGFP in neural crest cells and N- cadherin mismatch (MM) MO in placode cells, followed by immunostaining for Tubb3 (to label all neurons). Our results indicated that MO-positive cells are Tubb3-positive but GFP-negative, demonstrating that our N-cadherin MO only targets placode cells (new Supplemental Figure 4). We hope that this experiment addresses any concerns on the part of the Reviewer with regards to specificity of MO targeting.

2. The Reviewer writes "given the technique used [i.e., explant system], these data [Fig. 6] (and others in the paper) support tissue level autonomy of function, but not necessarily cell level autonomy (i.e. at an individual cell by cell level). The text - including the abstract - should be re-written to reflect this."

We appreciate the Reviewer's comment regarding use of the term "tissue level of autonomy" vs. "cell level autonomy" given the explant assay we are using. In perusing the literature, however, we found that articles with similar types of explant experiments, coupled with *in vivo* assays like we have performed, consistently use the phrase "cell" or "non-cell" autonomous vs. "tissue" autonomous. One example of this is Sanchez-Camacho and Bovolenta, 2008 (published in *Development*), which used explant cultures and *in vivo* electroporations of mouse embryos to parse out the role of Shh in retinal ganglion axon outgrowth and guidance. In Van Ho et al., 2011 (*Developmental Cell*), somite explants cultures and *in vivo* mouse embryo studies discerned non-cell autonomous effects on the balance of muscle progenitors vs. differentiating cells. Given this basis in the literature and our complementary *in vivo* assays, we have left the terms "cell autonomous" and "non-cell autonomous" in the text. However, if the Editor agrees with the Reviewer and would like us to modify the text, we would be happy to do so.

3. The Reviewer writes "the authors should briefly discuss the placode/crest interactions that occur in the Xenopus 'chase and run' study from Theveneau and Mayor as N-Cad is involved here too - albeit in a repulsive context."

We thank the Reviewer for pointing out this seminal work and apologize for its omission in our manuscript. We have now incorporated information on this publication in the manuscript.

© 2025. Published by The Company of Biologists under the terms of the Creative Commons Attribution License (https://creativecommons.org/licenses/by/4.0/).

Minor comment
The Reviewer notes that "Fig. 3A is missing a scale bar."

We apologize for the omission of this scale bar and have now added it to the figure.

Reviewer 3

1. The Reviewer asks us to "clarify how [we] ensured single cells were counted (if [we] did so) [in Figure 4]? Given that the quantitative analyses performed are based on neurites from a single soma, it is critical to ensure the authors are accounting for single cell bodies, especially because this experiment supports the non-cell autonomous function the authors posit for N-cadherin."

We apologize for not clarifying in more detail our methods for this experiment. To ensure single cells were counted, we went through each individual Z plane image in the stack and treated each separately to identify individual cells. We have added this information to the Materials and Methods section and hope this satisfies the concerns of the Reviewer.

2. The Reviewer writes the "there are no data showing what the 24h explants [for Figure 5] look like prior to addition of the antibodies. Without seeing analysis of axon density and/or length measurements prior to antibody addition, can the authors exclude the possibility that N-cadherin inhibition induces axon pruning, rather than just defects in axon outgrowth/branching? A representative image of the explant prior to antibody treatment and similar quantitative analyses added to the figure would clarify this point."

We agree with the Reviewer that this is an important issue to consider in our studies. To address this, we have analyzed wildtype trigeminal ganglia explants after 24 hours in culture (pre-treatment control explants), allowing us to establish/evaluate baseline axon outgrowth and the possibility of axon pruning in our other samples (rat IgG control, anti-N-cadherin). While the amount of Tubb3 fluorescence between pre-treatment explants (grown for 24 hours) and anti-N-cadherin-treated samples (grown for 48 hours) was similar, we observed a 60% reduction in Tubb3 fluorescence in the anti-N-cadherin-treated samples compared to rat IgG-treated samples (grown for 48 hours, see Figure 5E). These results point to decreased axon density in trigeminal ganglion explant cultures after blocking N-cadherin-based adhesion. Furthermore, intersection analyses assessing axon branching revealed that explants treated with anti-N- cadherin exhibited a significant increase in axonal branching compared to pre-treatment controls up to 1400 µm (Figure 5F). In contrast, rat IgG-treated explants exhibited a significant increase in branching up to 1750 µm compared to the pre-treatment group (Figure 5F). However, when comparing the anti-N- cadherin- and rat IgG-treated explants, significant differences were only observed up to 700 µm (Figure 5F).

Interestingly, of the neurons that did extend axons, the mean segment length of detected axons was similar between pre-treatment controls and anti-N-cadherin-treated explants, while the rat IgG-treated group had a decreased average segment length compared to both (Figure 5G). This observation for the rat IgG-treated group further supports the conclusion that increased axon branching is occurring over time. To verify this and attempt to rule out a role for axon pruning in causing this decreased segment length, all detected segments were visualized using a violin plot to ascertain the distribution of the data (Figure 5H). This confirmed an increase in smaller segments in the rat IgG-treated group, validating that the observed decrease in mean segment length (Figure 5G) was due to averaging long and short segments, and not of axons retracting over time and/or axon pruning. Collectively, these findings suggest N-cadherin-mediated adhesion promotes axonal outgrowth and complexity in trigeminal neurons *in vitro* but does not likely function in axon pruning. We thank the Reviewer for suggesting this experiment since the new data it has provided have greatly improved the manuscript.

Minor comments
1. The Reviewer writes "Figure 3 is missing a scale bar."

We thank the Reviewer for pointing this out and have now added this to the figure.

© 2025. Published by The Company of Biologists under the terms of the Creative Commons Attribution License (https://creativecommons.org/licenses/by/4.0/).

2. The Reviewer writes "Figure 4B has black text on the image [which makes it] difficult to read."

We apologize for this lack of clarity and have revised the image to make it easier to read.

3. The Reviewer comments that "the asterisks on some of the graphs (Fig. 2C,H; Fig. 5E) are very small and difficult to see."

We thank the Reviewer for this comment. We have now removed the asterisks representing significance from the figure because, upon making them bigger, they were numerous and overcrowding the figure. The asterisks (and corresponding significance levels) have now been moved to the figure legends.

We wish to express our thanks to you and the Reviewers for providing these highly constructive comments that have clarified the last remaining aspects of the manuscript. With these additional modifications, we believe that the manuscript is now acceptable for publication. We look forward to hearing from you soon and again thank you for handling our paper.

Second decision letter

MS ID#: dev.204369R1

MS TITLE: N-cadherin facilitates trigeminal sensory neuron outgrowth and target tissue innervation

AUTHORS: Caroline A. Halmi; Carrie E. Leonard; Alec T. McIntosh; Lisa A. Taneyhill

I have now received all the referees reports on the above manuscript, and have reached a decision. The referees' comments are appended below, or you can access them online: please go to .

The overall evaluation is positive and we would like to publish a revised manuscript in Development, provided that the referees' comments can be satisfactorily addressed. There are just a few minor points to address. Please attend to these in your revised manuscript and detail them in your point-by-point response. If you do not agree with any of their criticisms or suggestions explain clearly why this is so. If it would be helpful, you are welcome to contact us to discuss your revision in greater detail. Please send us a point-by-point response indicating your plans for addressing the referees' comments, and we will look over this and provide further guidance.

Reviewer 1

Advance summary and potential significance to field

See prior review.

Comments for the author

The authors did a thorough job of addressing all the comments from the previous review. Just a couple small suggestions on the new data presented. In Figure 4, the B-catenin expression is a nice addition. It would be useful to present the individual fluorescent channels here so that the B-catenin expression is clearly visualized. Also, in Supplemental Figure 3, trkA and trkB expression is nicely illustrated and shown to be not significantly changed when normalized to area. However, it does seem the distribution of the staining may be different. Please add to the manuscript if this is true across samples.

Reviewer 2

© 2025. Published by The Company of Biologists under the terms of the Creative Commons Attribution License (https://creativecommons.org/licenses/by/4.0/).

The authors have done a thorough job of responding to reveiwer comments. I am now comfortable that the manuscript is ready for publication.

Reviewer 3

Advance summary and potential significance to field

This manuscript examines the role of N-cadherin during trigeminal ganglion development. The neurons of this cranial ganglion are both placode- and neural crest-derived, with the placode-derived neurons being the first to send out axons. Taking advantage of the chick embryo, the authors demonstrate in vivo and in vitro that N-cadherin is required for axon outgrowth in both placode- and neural crest-derived neurons cell autonomously and non-cell autonomously. This work provides a significant conceptual advance to our understanding of gangliogenesis during early nervous system development because it establishes a sustained role for N-cadherin during multiple stages of trigeminal ganglion development, as well as provides evidence for the role of cell adhesion molecules in pioneer neurons.

Comments for the author

The authors addressed all my prior concerns (Reviewer 3) satisfactorily.

Second revision

Author response to reviewers' comments

We wish to thank you and the reviewers for your consideration and comments regarding our resubmitted manuscript entitled “N-cadherin facilitates trigeminal sensory neuron outgrowth and target tissue innervation” (Manuscript ID#: dev.204369). We are pleased that all the reviewers liked the revised manuscript, and as requested by them and yourself, we have revised the manuscript according to their comments. Changes made to the manuscript text are highlighted in yellow.

Reviewer 1

The Reviewer writes “it would be useful to present the individual fluorescent channels here so the B- catenin expression is clearly visualized” and to comment on potential differences in “the distribution of the [TrkA and TrkB] staining” in Supplemental Figure 3.

We thank the Reviewer for providing these suggestions. We have now updated Figure 4 with these individual channel images as requested. We also agree that there could be some subtle changes in TrkA and TrkB subcellular distribution upon N-cadherin knockdown in placode cells. While quantifying any of these potential changes is beyond the scope of this current manuscript, we have commented on this observation in the context of the Discussion. We hope the Reviewer finds these modifications acceptable.

We wish to express our thanks to you and the Reviewers for providing these highly constructive comments that have clarified the last remaining aspects of the manuscript. With these additional modifications, we believe that the manuscript is now acceptable for publication. We look forward to hearing from you soon and again thank you for handling our paper.

Third decision letter

MS ID#: dev.204369R2

© 2025. Published by The Company of Biologists under the terms of the Creative Commons Attribution License (https://creativecommons.org/licenses/by/4.0/).

MS TITLE: N-cadherin facilitates trigeminal sensory neuron outgrowth and target tissue innervation

AUTHORS: Caroline A. Halmi; Carrie E. Leonard; Alec T. McIntosh; Lisa A. Taneyhill
ARTICLE TYPE: Research Article

I am happy to tell you that your manuscript has been accepted for publication in Development, pending our standard publication integrity checks.

© 2025. Published by The Company of Biologists under the terms of the Creative Commons Attribution License (https://creativecommons.org/licenses/by/4.0/).
